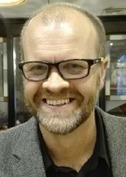# Electroactive Amphiphiles for Addressable Supramolecular Nanostructures

**DOI:** 10.1002/cnma.201800194

**Published:** 2018-07-04

**Authors:** E. J. Townsend, M. Alotaibi, B. M. Mills, K. Watanabe, A. M. Seddon, C. F. J. Faul

**Affiliations:** ^1^ School of Chemistry University of Bristol Cantock's Close Bristol BS8 1TS UK; ^2^ Bristol Centre for Functional Nanomaterials H.H. Wills Physics Laboratory University of Bristol Tyndall Avenue Bristol BS8 1TL; ^3^ Chemistry Department Faculty of Science King Abdul Aziz University Jeddah, KSA; ^4^ Research Organization of Science and Technology Ritsumeikan University 1-1-1 Noji-higashi Kusatsu, Shiga 525-8577 Japan; ^5^ School of Physics H.H. Wills Physics Laboratory University of Bristol Tyndall Avenue Bristol BS8 1TL

**Keywords:** amphiphiles, functional nanostructures, molecular electronics, redox active, self-assembly

## Abstract

In this focus review we aim to highlight an exciting class of materials, electroactive amphiphiles (EAAs). This class of functional amphiphilic molecules has been the subject of sporadic investigations over the last few decades, but little attempt has been made to date to gather or organise these investigations into a logical fashion. Here we attempted to gather the most important contributions, provide a framework in which to discuss them, and, more importantly, point towards the areas where we believe these EAAs will contribute to solving wider scientific problems and open new opportunities. Our discussions cover materials based on low molecular weight ferrocenes, viologens and anilines, as well as examples of polymeric and supramolecular EAAs. With the advances of modern analytical techniques and new tools for modelling and understanding optoelectronic properties, we believe that this area of research is ready for further exploration and exploitation.

## 1 Introduction

Early reports describe the concept of self‐assembly as the spontaneous arrangement of molecules into stable and well‐defined structures under equilibrium conditions.^[1]–[3]^ Initial research into self‐assembled systems and structures exploited a range of noncovalent interactions (including hydrogen bonding,^4^ electrostatic,^5^ stacking^6^ and other soft van der Waals interactions^7^) and generated constructs with nanoscale dimensions. Researchers continued to explore self‐assembly as a strategy to create (soft) nanostructures, as discussed in various previous reviews.^8^ –^10^ As the field has developed and matured, recent advances have shown that it is now possible to use self‐assembly to control structures over multiple length scales, and to prepare well‐defined three‐ dimensional (3D) objects.^11^

With this level of control over structure now possible, current challenges include extending this level of control to the function of self‐assembled structures. Applications of such assembled functional constructs are emerging in a number of important areas: for example, we showed recently that the ability to control the length of well‐defined self‐assembled fiber‐like micelles can be utilized to control function in simple OFET devices.^12^ Carefully designed peptide‐based materials have shown potential applications as smart devices for biomedical applications.^13^,^14^ Tuning the self‐assembled structures in supramolecular materials provides a new strategy to improve the performance in cancer diagnostics and therapy, opening applications in the field of nanomedicine.^15^,^16^ Nanostructured soft materials show further attractive properties, including self‐healing,^17^ self‐regulation, emergent self‐replication^18^ and artificial enzyme functionality.^19^ These properties make these materials particularly suitable for bioelectronic applications.

One of the classic, most well‐known and studied classes of self‐assembling materials are amphiphiles. Both low Mw and polymeric, high Mw amphiphiles form various well‐defined structures such as spherical or worm‐like micelles or vesicles in solution. The self‐assembled structures depend on the chemical nature and architecture of the amphiphiles, the latter of which is defined by a geometric parameter, the packing parameter p . The packing parameter can be used to predict the structure of the aggregate using p=v /$a_{0}l_{c}$ , where v is the volume of the hydrophobic chain, $l_{c}$ is the critical chain length, and $a_{0}$ is the surface area of the hydrophilic headgroup. Changing these parameters, spherical $\left(p\leq \frac{1}{3}\right)$ , cylindrical ($\frac{1}{3}<p<\frac{1}{2})$ , vesicles or flexible bilayers ($\frac{1}{2}\leq p<1$ ), lamellar $\left(p=1\right),\;$ and even inverted $\left(p>1\right)$ structures can be accessed.^20^ This fundamental and widely used concept is of particular importance for our discussions here as changes to the individual terms of the packing parameter will lead to significant changes in assembled structures and properties.

Although amphiphiles (whether simple, bola or gemini amphiphiles, low or high Mw) are widely used, an interesting subset of these materials seem to have only received sporadic attention, with little attempt at any form of classification, in the last few decades. This subset, which constitutes of and is defined by amphiphiles functionalised with or containing moieties that exhibit redox‐switchable behaviour (e. g., viologens, ferrocenes or oligo(aniline)s), are described here as electroactive amphiphiles (EAAs). These functional self‐assembling systems are the focus of this review article.^21^

There are a number of review papers that cover related aspects and topics that are not discussed here, including those focused on electrochemically controlled supramolecular systems,^22^ functional oligo(thiophene)‐based materials,^23^ self‐assembled gelators and their optoelectronic applications,^9^ functional and bolaamphiphilic systems and their potential applications,^24^,^25^ controlled self‐assembly of biomolecules functionalised with π‐conjuated oligomers,^26^ self‐assembly of supramolecular amphiphiles including some electoactive systems^8^,^27^, ^28^, ^29^, ^30^, ^31^ and some conjugated polymeric amphiphilic systems,^25^ amongst others.

**Scheme 1:**
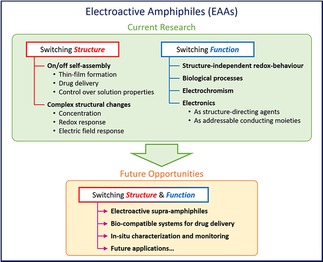
Overview of the classification of EAAs presented in this review.

Hence, in this review we aim to provide a simple classification of EAAs and cover the literature to show existing examples. The manuscript is structured into three main sections, as shown in Scheme 1: a) switching structure, b) switching function and c) future opportunities, with the main focus on reported papers that exhibit strategies to tune either structure or function and, importantly, attempt to highlight the appropriate applications. We will also point to the many opportunities related to EAAs and topics of current interest in the wider field of functional materials chemistry: functionality, switchability, tuneability and addressability, and attempt to highlight novel applications.

## 2 Switching Structure

The first and most basic category to be discussed are EAAs that undergo a change in morphology with the application of an external stimulus, here an oxidising/reducing agent or applied voltage. The structural change may either be a simple transition from aggregate to disassembled monomer, or a more complex structural transition such as from a spherical micelle to a nanorod. These structural switches will be discussed below along with some proposed relevant applications.

### 2.1 On/off Self‐assembly

One of the most widely studied molecules for reactive self‐assembly is ferrocene.^32^ In its reduced state ferrocene exists as the relatively non‐polar neutral Fe(II) species. However, it can be easily oxidised into the polar ferrocenium cation (Fe(III)). This change can lead to redox‐sensitive behaviour and changes in water solubility of any molecule containing this moiety. A wide range of ferrocenyl‐derived EAAs have been reported, from single‐ and double‐tailed surfactants^32^,^33^ to amphiphiles complexed with other molecules,^34^, ^35^, ^36^ all of which exhibit notable changes in self‐assembled morphologies with change in oxidation state. An attractive example comes from Alkan *et al*.^37^ who synthesised a ferrocene block co‐polymer from ethylene glycol and a ferrocenyl glycidyl ether that formed micelles in aqueous solutions. In the reduced form, the micelles were stable for months. However, when oxidised the micelles rapidly dissolved as the formation of the ferrocenium cation led to a polymer with two hydrophilic moieties.

Another commonly used electroactive unit is tetrathiafulvalene (TTF). TTF is a π‐electron‐donating molecule with three stable oxidation states that can be easily and reversibly accessed. Additionally, the presence of π–π and sulfur–sulfur interactions allows TTF and its derivatives to self‐assemble into complex nanostructures.^38^ As with ferrocene, TTF has been coupled with hydrophilic moieties to prepare redox‐responsive aggregates that can be disrupted by the addition of an oxidant.^39^ Disassembly occurred as the TTF moiety was oxidised to the more hydrophilic radical cationic and dicationic states, which, in turn, induced electrostatic repulsion between amphiphiles. The aggregate morphologies (i. e., before disassembly) were also dependent on the molecule's shape, as a dumbbell‐shaped amphiphile formed vesicles, while wedge‐shaped amphiphiles formed smaller micelles.^40^ This work highlights the importance of molecular shape as well as chemical functionality when designing amphiphiles for specific applications, factors that will be considered again in the final parts of this review.

There are many possible uses for targeted self‐assembly, mainly focused on controlled uptake and release of a hydrophobic substance that can be protected within and delivered by an EAA. Ferrocenyl‐based amphiphiles that form micelles or vesicles are often investigated for the solubilization of hydropholic substances and have been shown to release loaded materials depending on the degree of the amphiphile's oxidation.^37^,^41^, ^42^, ^43^

**Figure 1:**
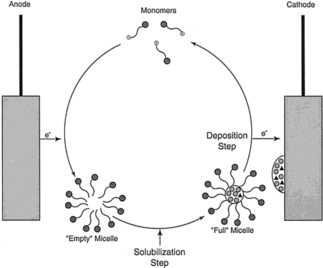
Schematic illustration of a separations scheme based on the electrochemical assembly and disassembly of micelles. Reproduced with permission from Ref. 44.

Before looking in more detail at specific applications, it is worth mentioning a very simple but elegant system discussed by Rosslee and Abbott.^44^ They suggested using a ferrocenyl EAA (11‐ferrocenylundecyl)trimethylammonium bromide) for the microscale separation of hydrophobic compounds (Figure 1). The EAA in its oxidised form is added to a solution containing a mixture of compounds. When reduced, the EAA forms micelles, solubilising the hydrophobic compounds. The contents can then be released by re‐oxidation. In this manner the authors demonstrated selective solubilization and deposition of dyes from a mixed solution. However, this process is slower than chromatography or electrophoresis, so the authors suggested that the EAA would be more appropriate for separation, where speed is less crucial than in analysis. In addition to this strategy for separation, a number of other applications have been explored where on/off assembly is of importance. These are briefly discussed here.

#### 2.1.1 Thin‐film Formation

A simple application for EAAs that demonstrate controlled release of materials is the formation of thin films. This strategy involves using an amphiphile to solubilise a desired material into a micelle or vesicle. The self‐assembled aggregate then carries the compound to the substrate of interest, where a stimulus is used to disassemble the aggregate into its constituent monomers and release the contents. The released substance then forms a thin film on the substrate surface. A proof‐of‐concept study demonstrated the use of a micelle‐forming ferrocenyl‐based surfactant to contain and release organic pigments, forming thin films of the dye at an electrode's surface.^45^ The authors suggested that this technique is advantageous in comparison to films produced by electrochemical reactions as the compound forming the film is not chemically altered by the deposition process.

#### 2.1.2 Drug Delivery

Another important area for targeted release is in drug delivery, with the aim to increase the concentration of the desired drug at the affected area of the body while limiting release in other areas. Targeted delivery is of particular importance when the drug is toxic or has undesirable side‐effects.

A class of EAAs that has the potential to be used for targeted delivery are the oligo(aniline)s.^46^ Oligo(aniline)s are generally classified as biocompatible^47^, ^48^, ^49^ and can exist in multiple oxidation states; the leucoemeraldine base (LEB), emeraldine base (EB) and pernigraniline base (PB). The states can be reversibly accessed with the application of an oxidising or reducing voltage. Wu *et al*.^50^ have developed polymers prepared from tetra(aniline), TANI, covalently linked with poly(N‐isopropylacrylamide) (PNIPAM). These amphiphilic moieties formed large vesicles with multiple bilayer structures that could be loaded with the anti‐cancer drug doxorubicin. Oxidation to the EB state caused the vesicles to be destroyed, releasing the drug. The amphiphiles displayed orthogonal switchability, as in addition to responding to voltage, the incorporation of PNIPAM allowed the amphiphile to respond to changes in pH and temperature.

An alternative drug‐delivery system was developed by Yan *et al*.^36^ who used the host‐guest interaction of a β‐cyclodextrin‐derived polymer and ferrocenyl polymer to prepare vesicles that reversibly dissociate with the application of an external voltage. The authors showed the polymers formed aggregates of approximately 100 nm in radius and were stable for months until electrostimulation. Interestingly, the rate of dissociation and thereby drug release were dependant on voltage, with drug release occurring after 30 minutes for a +4.0 V applied voltage and 7.5 hours for a +1.0 V applied voltage. This approach could provide an avenue for highly sensitive drug delivery systems.

Triggered release may not always be possible for areas of the body that are not accessible by external stimuli. In those situations, amphiphiles that undergo time‐dependent changes might be an attractive option. A polymeric EAA was prepared by copolymerisation of poly(ethylene glycol)‐co‐poly(lactide) with an aniline pentamer.^51^ In the LEB state, the EAA formed micelles between 30 and 60 nm in diameter. However, the LEB state was not stable in air and oxidation occurred over time, producing the EB state. The presence of the EB state led to an increase in the critical micelle concentration (CMC) of the EAA, with consequent dissociation of the micelles. Oxidation took place over a matter of days, which may limit the applications for drug delivery. However, further developments would be required to also consider *in vitro* and *in vivo* rates of oxidation.

Most researchers have focused on EAAs that display reversible behaviour; however, for some applications reversibility is not necessary, for example in the case of drug delivery or thin film deposition. In such cases, Jacob *et al*.^52^ suggested the use of a ruthenocene‐based amphiphile. Ruthenocene has attracted much interest as it is relatively non‐toxic, can exist in multiple oxidation states, and may be used as a pro‐drug.^53^,^54^ For example, ruthenocene complexes used in chemotherapy may only become activated in the reducing environment of tumour cells, limiting toxicity in other parts of the body;^54^ it is important to note that ruthenocene exhibits irreversible electrochemistry.^55^ Jacob *et al*.^52^ showed that the EAA dodecyl‐dimethyl(methylruthenoceyl) ammonium bromide forms micelles in aqueous solution when above the CMC (0.4 mM). The micelles were unstable when electrochemically oxidized, which allowed the release of water‐insoluble materials from the micelles.

Finally, researchers are investigating the use of redox agents to trigger disassembly in place of an applied voltage.^[56],[57]^ Such systems could use reducing or oxidising agents found inside the body to trigger release which would negate the need to implant a voltage source.^58^ For example, Zhao *et al*.^57^ prepared a supramolecular assembly from the host‐guest interaction between cucurbit[8]uril and methyl viologen. When the reducing agent sodium dithionite was added, the assemblies gradually increased in size from 176.8 nm to 1 μm which allowed the loaded drug to be released.

#### 2.1.3 Control over Solution Properties

Aside from targeted release, EAAs were also investigated for potential control of fluid properties such as viscosity and surface tension. Tsuchiya *et al*.^59^ described the development of an electroresponsive fluid prepared from a ferrocenyl‐based EAA (11‐ferrocenylundecyl)‐trimethylammonium bromide), which forms micelles in the presence of sodium salicylate. When the ferrocenyl group was electrolytically oxidised, the micelles dissociated into monomers and the solution viscosity decreased to several orders of magnitude below that of the reduced form. The authors suggest this system could be used for responsive fluids in printers, dyes and perfumes.

The same EAA was also used to control the surface tension of a liquid.^60^ The results were dependent on the oxidation state and concentration of the amphiphile. At concentrations close to the CMC of 0.1 mM, oxidation led to an increase in surface tension, while at concentrations much higher than the CMC, oxidation led to a decrease in surface tension. This behaviour was ascribed to an increase in the hydrophilicity of the EAA, which at concentrations below the CMC leads to desorption from the surface, increasing the surface tension. Alternatively, at concentrations greater than the CMC, oxidation caused micelles to disassociate in the bulk solution, increasing the chemical potential and surface concentration of the EAA, thus decreasing the surface tension.

### 2.2 Complex Structural Changes

The examples above describe simple “on/off” behaviour, where the EAA is either in a monomeric or aggregated form depending on the oxidation state. However, there are many examples of EAAs that show more complex structural changes with a change in oxidation state, including those that can be oriented owing to the presence of their electroactive component. Such complex behaviour could be important where properties are enhanced or diminished by a change in morphology, making them potentially suitable for a wider range of applications.

#### 2.2.1 Concentration

Although the most commonly investigated structures for EAAs are micelles and vesicles, it is possible to prepare other structures. Kakizawa *et al*.^42^ synthesised a double‐tail EAA with two ferrocenyl moieties, with the aim of providing reversible control over the formation of micelles for drug delivery. They found that the morphology was concentration dependent and ranged from dissolved molecules to micelles and finally liquid crystals with increasing concentration. Although this behaviour did not originate from an electroactive response, it is important to consider as the properties of the aggregates were highly dependent on the morphology, which depended on the oxidation state. The authors measured the solution conductivity as the concentration of the EAA was increased and the dissolved monomer formed vesicles. The transition was associated with a decrease in specific conductivity, which was attributed to the separation of counterions in the aqueous phase by the alkyl bilayer.

#### 2.2.2 Redox Response

**Figure 2:**
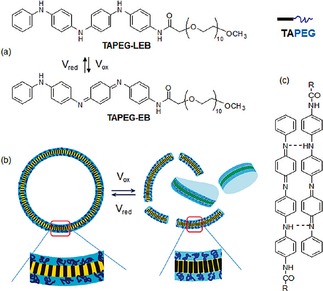
Voltage‐responsive TAPEG rod‐coil EAAs in aqueous solution. (a) Chemical structures of TAPEG in the LEB and EB oxidation states. (b) Schematic representation of redox switching between vesicles and puck‐like micelles induced by the change in packing density in the membrane core. (c) Amine‐imine intermolecular hydrogen bonding in the EB form of TAPEG. Reproduced with permission from Ref. 61.

It is also possible to control the overall morphology of assemblies by altering their redox state (i. e., not just a simple on/off response). Some particularly attractive examples are based on oligo(aniline)s. As mentioned in **Section 2.1.2**, oligo(aniline)s can exist in multiple oxidation states and can be interconverted with a chemical oxidant or oxidising voltage. A rod‐coil EAA, derived from a TANI core and poly(ethylene glycol) tail (abbreviated TAPEG), formed vesicles in the LEB state (∼8 nm in diameter, Figure 2).^61^ When an oxidising voltage was applied, the vesicles split into smaller puck‐like micelles (∼4 nm in diameter). This change was attributed to a change in packing behavior, which was influenced by the formation of amine–imine hydrogen bonds in the EB state. The authors postulate that such molecules are preferable for drug delivery systems as simple structural rearrangements between two soluble species would avoid potential problems with lowered solubility and thus possible *in vivo* precipitation of the carrier.

**Figure 3:**
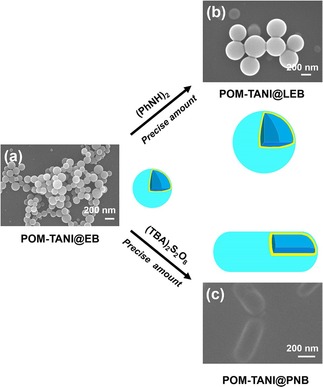
SEM images of the formed self‐assembled structures in different oxidation states: a) POM‐TANI@EB. b) POM‐TANI@LEB. c) POM‐TANI@PNB. Reproduced with permission from Ref. 62.

A system showing similar behaviour was developed by She *et al*.^62^ from a polyoxometallate (POM) coupled with a covalently linked tri(aniline). Although the structure here might not fit with that of a classical EAA, the assembled system adapted different morphologies depending on the oxidation state of the tri(aniline). Oxidation from the EB state to the PB state changed the morphology of the EAA from micelles to nanocapsules (Figure 3). Alternatively, when reducing from the EB state to the LEB state, the vesicles increased in size from a diameter of 200 nm to 250 nm. The self‐assembled structures could be used to prepare surfaces with tuneable wettability, as the different morphologies had different surface energies (and thus led different contact angles with liquids).

Further EAAs that demonstrate very elegant orthogonal switchability have also been prepared (see also **Section 2.1.2 Drug delivery**). Specifically, an EAA based on anthraquinone, AQUA (AQNH(CH_2_)_10_COOH, where AQ is anthraquinone), formed nanotubes in water in the presence of an equimolar amount of ethanolamine.^63^ Anthraquinone, a redox‐active group, can reversibly transition from a poor to a good conductor after reduction.^64^ When chemically reduced, the nanotubes transformed into thin ribbons and exhibited an increase in conductivity. This transition could be reversed by the addition of an oxidant. The morphology of the aggregates could also be affected by changing the pH, which addressed the carboxylic acid head group of the amphiphile. At pH values below the pK_a_ of the COOH group, the nanotubes deformed into flatter, open sheets. Interestingly, the response time of the pH‐induced transition was only 30 minutes, which was much faster than the very long oxidation/reduction transitions (90 hours), with no further explanation provided for this intriguing behaviour.

#### 2.2.3 Electric Field Response

**Figure 4:**
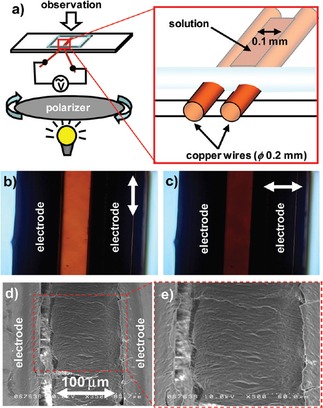
(a) Schematic illustration of the experimental cell for investigating optical anisotropy of coordination nanowires under applied ac electric field (b, c) Polarizing microscope images of decane solution of 1 (X=Cl): (b) perpendicular to the direction of electric field, (c) parallel to the applied electric field. (d, e) Scanning electron micrographs of 1 (X=Cl) immobilized between opposing electrodes. Reproduced with permission from Ref. 65.

The electroactive properties of EAAs can also be used to address and to orientate the overall assembled structures on a large (microscopic or even macroscopic) scale. Kuwahara *et al*.^65^ prepared amphiphilic diruthenium complexes that formed columnar liquid crystal structures when bridged using a halogen counterion in organic media. The aim of the study was to prepare complexes that would orientate on a macroscopic level into nanocircuits. The halogen counterion (Cl, I) had considerable influence on the self‐assembly and alignment of the complexes (when applying an electric field; see Figure 4 for the experimental setup). Chloro‐bridged complexes aligned slowly and were stable to increased temperature when applying an external alternating electric field. Alternatively, iodo‐bridged complexes rapidly and reversibly aligned.

## 3 Switching Function

### 3.1 Structure‐independent Redox Behaviour

Many electroactive materials will undergo changes in oxidation state without a corresponding structural change. For these materials, their electroactive properties play a vital role in preparing materials with switchable functionality, for example materials with tuneable conductivity, or materials to be used in electrochromic devices or in biomedicine. The self‐assembly of these EAAs allows for the preparation of well‐defined structures, with properties that are often enhanced owing to the macroscopic order of the material. A wide range of such EAAs have been investigated, from small molecules such as anthraquinone and POMs,^66^,^67^ to oligomers^68^ and polymers.^69^,^70^

### 3.2 Biological Processes

EAAs could play an important role in controlling biochemical processes, as these moieties are often non‐toxic and water‐soluble. Many biological processes involve redox reactions and thus EAAs have already been investigated for use in a wide range of research areas. For example, EAAs that self‐assemble into fibrillar network structures have been used as scaffolds to facilitate nerve regeneration and promote more advanced neural differentiation than nonconductive materials.^71^ In a different approach, ferrocene‐containing EAAs have been used to mimic cationic lipids that can transfer DNA across cell membranes, promoting gene expression. In the reduced state, the EAA promotes high cell transfection, while the oxidised state shows very low levels.^72^ Additionally, EAAs have been used to model membrane systems, demonstrating how redox‐active materials can partition into a lipid bilayer.^73^

Another area of interest involves controlling the reaction rates within cells. Cellular metabolism is controlled by the internal redox environment of the cell, which can also be controlled by the addition of an electroactive material. Recently, Kaneko *et al*.^74^ developed an amphiphilic, cell‐membrane‐permeable polymer from the polymerization of 2‐methacryloyloxyethyl phosphorylcholine and vinyl ferrocene. They demonstrated the polymeric EAA's effectiveness at mediating reaction rates in a cell by acting as an electron acceptor, leading to enhanced cell growth.

### 3.3 Electrochromism

**Figure 5:**
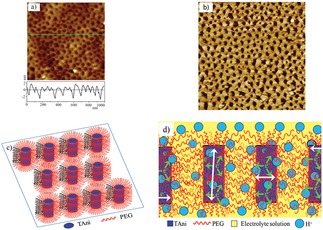
AFM image (a) and phase contrast of (TAni‐*b*‐PEG)3 copolymer thin film Schematic illustrations of c) the self‐assembly of (TAni‐*b*‐PEG)3 thin film, and d) ion and electron diffusion pattern in (TAni‐*b*‐PEG)3 thin film in HCl electrolyte solution. Reproduced with permission from Ref. 75.

Electrochromic materials are materials where the application of a voltage will lead to reversible changes in their optical properties. There is a growing market for electrochromic materials, for example, in smart windows, displays and sensors. For these applications it is useful to have well‐defined, easily processable and addressable materials such as EAAs. Oligo(aniline)‐derived amphiphiles have received a great deal of attention for the preparation of such devices. Each oxidation state has a distinct associated colour, making such EAAs very attractive as electrochromic materials. Cao *et al*.^75^ synthesised a star‐shaped molecule consisting of a benzene core and three TANI‐polyethylene glycol arms. The EAA formed micelles in solution which allowed the amphiphile to assemble into a highly‐ordered film when cast onto an indium tin oxide (ITO) substrate (Figure 5). The film showed enhanced electrochromic properties that were attributed to the self‐assembled structure producing pathways for fast electronic transfer.

A further class of stable and well‐studied electrochromic materials are POMs. The visible absorption band in POMs originates from the ligand‐to‐metal charge transfer, allowing the optical response to be tuned by ligand type.^76^ Although these materials are not considered amphiphilic, POMs can be complexed with EAAs to produce aggregates with interesting electroresponsive behaviour. Zhang *et al*.^77^ prepared europium‐based POM complexes formed with different amphiphilic groups (ferrocenyl amphiphiles and single, double and triple alkyl bromide tails). The amphiphiles act as a structure directing agent for the POM, inducing the formation of POM‐surfactant complexes with lamellar structures that display thermotropic and lyotropic behaviour. Most complexes demonstrated reversible redox and electrochromic behaviour, transforming from transparent to blue or blue/purple with increasing negative potential. However, the ferrocene‐containing compounds showed no electrochromism, which was attributed to an electron‐transfer interaction between the POM anion and the ferrocenyl moiety. Although no changes in assembly were noticed, Zhang *et al*.^78^ showed that the presence of a ferrocene‐containing amphiphile used to construct soft nanostructured materials could be used to quench the electrochromism of POM‐based LC materials. The presence of viologen‐containing amphiphiles ensured intensely coloured assembled structures (when compared with materials prepared from simple alkyl chain amphiphiles) and was attributed to the formation of the strongly coloured radical cations of the viologen.

### 3.4 Electronics

The most common area where EAAs are envisaged to find application is in organic electronics. Since the discovery of metallic conductivity of polyacetylene by Shirakawa *et al*. in 1977,^79^ organic molecules with delocalised π‐electron systems have been extensively investigated for electronic devices. The ability of EAAs to self‐assemble provides ways to control macroscale order, which in turn allows for the minimisation of packing defects and domain boundaries, thus increasing the overall charge carrier mobility.^80^ Two main areas of investigation within this topic have been EAAs as structure‐directing agents for conductive materials, or as the actual conductive unit.

#### 3.4.1 EAAs as Structure‐directing Agents

Polythiophenes (PTs) have attracted attention in the production of solar cells owing to their relatively high hole mobilities and absorption in the visible region. Blends of PTs with semi‐conductive nanoparticles such as CdSe have been used to prepare organic‐inorganic hybrid solar cells.^81^ Coating the nanocrystals with amphiphiles allows for enhanced solubility in nonpolar solvents, preventing aggregation. EAA coatings have the additional benefit of being able to mediate electron transfer between organic and inorganic materials at the surface/electronic interface.^82^

In addition to stabilising nanocrystals, polymeric EAAs based on carbazole have been used to disperse multi‐walled carbon nanotubes (MWCNTs) in water.^83^ The carbazole polymers self‐assemble into micelles on the surfaces of the MWCNTs, preventing aggregation by steric repulsion, which increased the overall conductivity of the material. Moreover, the electroactive polymer additionally allowed the formation of a conductive network between the MWCNTs to accelerate electron transfer.

EAAs can thus play roles as both a dispersion agent and a charge‐carrier mediator, and could therefore be promising candidates to improve the processability and conductivity of inorganic materials.

#### 3.4.2 EAAs as Addressable Conducting Moieties

**Figure 6:**
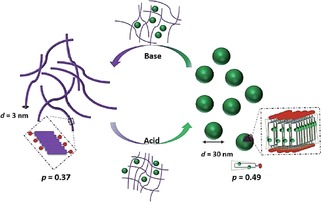
Schematic showing the proposed doping/de‐doping responsive self‐assembly behaviour of TANI(TFA)_2_‐PTAB in aqueous solution. Reproduced with permission from Ref. 87. Published by The Royal Society of Chemistry.

As discussed at the start of this section, conductivity often depends on the self‐assembled structure of the electroactive materials. The aim is therefore to utilize self‐assembly to improve the co‐facial π‐π interactions and overlap, thereby increasing charge carrier mobilities.^84^ Poly‐ and oligo(aniline)s are insulators when prepared in the LEB, EB or PB state. However, they can access a fourth conducting state, the emeraldine salt (ES) state, either by oxidative doping from the LEB state or by acid doping from the EB state. Recently, a water‐soluble TANI amphiphile (TANI‐PTAB) was prepared bearing a trimethylammonium headgroup. This EAA self‐assembled into nanowires in solution when in the EB state.^85^ The nanowires were conductive (2.7 mS cm^−1^) after doping with camphorsulfonic acid, which had the additional effect of inducing helicity dependant on the chirality of the dopant. The authors note that while the conductivity is not as high as that of crystalline TANI structures,^86^ the ease of preparation makes it more attractive for future electronic devices. These concepts were further exploited in a recent study, where dopants used to form the ES state were also used to tune the packing parameter, and thus the final assembled structure of this EAA.^87^ As the doping process is fully reversible, Lyu *et al*.^87^ showed that this strategy could be successfully applied to switch both assembled structure and optoelectronic properties of these EAAs, as shown in Figure 6.

An alternative approach to improving conductivity by adding dopants is to mix π‐electron donating and π‐electron accepting materials. Recently, Khalily, M. A. *et al*.^84^ prepared amphiphilic p‐type and n‐type peptides from pyrene and a naphthalenediimide. The peptides individually formed nanofibers but could also co‐assemble into nanowires. The overall conductivity was several orders of magnitude greater than the individual fibres.

In some cases, the self‐assembly of EAAs in solution is used to direct the formation of highly ordered thin films. For example, Raymo *et al*.^89^ prepared functional materials based on EAAs derived from the 4,4‐bipyridinium dication. The amphiphile could either form organised monolayers at air/water interfaces and transferred to the surfaces of electrodes using a Langmuir‐Blodgett technique, or would spontaneously adsorb on electrode surfaces from solution. In both cases the amphiphiles retained their electroactive behaviour and could be used in the design of molecule‐based devices.

The solvent used to prepare films is also important to consider when preparing electronic devices from EAAs. Kim *et al*.^89^ prepared a rod‐coil‐rod polymeric EAA consisting of TANI‐poly(ethylene glycol)‐TANI that formed vesicles in solution. However, when cast onto ITO substrates, the effective concentration of the EAAs increased and the molecules reorganised into highly ordered lamellar structures when cast from water, or more disordered structures when cast from THF. Interestingly, the films prepared from THF showed greater electrochemical activity than the multi‐layered lamellar films, owing to the interconnected microdomains produced.

Similarly, quinquethiophene‐derived gels were prepared that displayed different conductivities dependant on the solvent used for self‐assembly.^90^ When cast from cyclohexane the EAAs the gels were conducting on the same order of magnitude as polythiophenes deposited in the same manner. However, when cast from chloroform, conductivity was negligible. The authors postulated that an extended nanofiber network with hydrogen‐bond formation and π‐stacking is important for the observed high conductivity.

EAAs show a high degree of order and high conductivity when prepared in solution or cast into thin films, making them attractive materials for the preparation of soft electronic devices.

## 4 Future Opportunities

A number of opportunities for future development exist for this exciting and interesting class of self‐assembled functional materials – EAAs. We suggest that with a concerted effort from the research community, EAAs will be able to add value and address important issues related to controlled release (of drugs, especially related to cancer therapy, and of other active ingredients, for example in the agrochemical sector), as well as control over rheological properties of fluids, for example, in the printing of functional devices.

However, these developments will not be without their own challenges, as most of the current studies focus on one aspect (e. g., structural changes) rather than on changes in both structure and function. We believe that to unlock the potential of EAAs, combinations of redox‐addressable structure and function or inclusion of orthogonal addressability or function, should be carefully considered. An area that is undergoing very rapid and promising development is that of electroactive supra‐amphiphiles. Owing to the advantages provided through the facile supramolecular construction of such complex materials, we expect that this class of functional amphiphiles will continue to attract attention, especially for biocompatible systems for drug delivery.

In addition to these larger overarching issues and goals, some very specific challenges and opportunities exist. On a very fundamental level, in‐situ monitoring using combinations of structural and spectroscopic techniques could be employed to show time‐resolved structural evolution and how these structural changes impinge on the properties of these materials. X‐ray techniques such as small angle X‐ray scattering (SAXS) are commonly employed to probe the solution structure of self‐assembled molecules, for example the TANI‐based electroactive amphiphiles described in **Section 3.4.2**. However, they can potentially also be used to understand dynamic structural changes brought about by addition of dopants which is a potentially exciting future direction in which to take advantage of time‐resolved scattering sample environments.

Stopped‐flow techniques have been employed in a range of soft matter, colloid, and biomolecule studies to probe the early stages of kinetics of formation or organisation of materials.^91^ Advances in synchrotron X‐ray capability now mean that these kinetics can be probed on time scales on the order of 10 ms. The benefits of stopped‐flow apparatus are that the mixing of several components can be controlled precisely, and more importantly, that the synchronisation of the onset of mixing and the acquisition of the scattering pattern can be done in a reproducible manner. The short dead time of this technique would allow the capture of the early onset of assembly. One can therefore envisage using this technique to follow the changes in structure of, for example, the TANI‐based amphiphiles during doping and de‐doping, shedding light on the nature of their structural changes and following their evolution through to equilibrium. Given that interfacing stopped flow with other techniques, such as spectroscopy, is also relatively trivial, this opens up the possibility of measuring structural changes and electronic properties simultaneously in a single experiment.

A less common method than stopped flow, but which nevertheless has recently been employed in examining the dynamic changes of amphiphile self‐assembly, is acoustic levitation coupled with SAXS. Recent investigations^92^ showed that self‐assembling lipid and surfactant molecules can be levitated in a millimetre sized solvent droplet, and that the resultant self‐assembly behaviour can be captured using synchrotron SAXS, leading to data of comparable quality to that taken from capillaries. Furthermore, by changing the water content of the droplet environment, the phase behaviour of the amphiphile can be tuned. Further work by Pfrang *et al*.^93^ coupled these levitated droplets not only with SAXS, but also with Raman spectroscopy. Introduction of O_3_ into the droplets oxidised the amphiphiles (in this case, fatty acids) and caused structural rearrangement of the molecules, which was followed in real‐time, alongside the collection of Raman data, to monitor the breaking of the C=C bond in the fatty acid tail. This methodology could easily be adapted to a range of electroactive surfactants to investigate changes in properties and structure on the introduction of redox‐active reagents in the gas phase.

Certain classes of materials, such as the oligo(aniline)‐based materials, have recently undergone significant and promising developments – the expectation is that further exciting advances can be made with these versatile materials, though challenging synthesis of such materials could pose a barrier. Other, more common small molecule systems, such as the viologens, could also be explored further. Some papers discuss viologen‐derived amphiphiles, though they either focus on electroactivity or self‐assembly but not both. For example, Lin *et al*.^94^ discuss rod‐rod type amphiphiles using viologens as the hydrophilic segment and phenyls as the hydrophobic segment. The amphiphiles form different structures depending on the hydrophilic/hydrophobic balance. There is no mention of electroactive response. Alternatively, viologen amphiphiles have been used to prepare Langmuir‐Blodgett films,^95^ or for transport of dyes to an electrode (before desorption and deposition).^96^ Development of other soft functional systems for optoelectronic applications^97^ could also lead to applications as in‐situ sensors for monitoring the presence of radicals or redox‐active pollutants. Such systems are obvious areas for further investigation and development.

In conclusion we believe that the study of EAAs is about to enter an exciting era; the addition of novel theory and modelling to the range of approaches proposed here, will provide opportunities to develop design rules for a new generation of EAAs.

## Data Statement

No new data was generated for this paper.

## Conflict of interest

The authors declare no conflict of interest.

## Biographical Information

*Esther Townsend received her B.Sc. degree from Keele University. Currently, she is a Ph.D. candidate in the Bristol Centre for Functional Nanomaterials CDT at the University of Bristol, working under the joint supervision of Prof. Charl Faul and Dr Annela Seddon. Her research focuses on the directed self‐assembly of electroactive amphiphiles*.

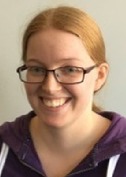

## Biographical Information

*Maha Alotaibi received her B.Sc and M.Sc in the department of chemistry at King Abdul Aziz University in 2010. She received a scholarship and joined Prof. Charl Faul in 2015, pursuing her PhD in Materials Chemistry. Her current research focuses on the preparation of oligomeric amphiphiles and investigates the ability to tune the self‐assembled structures they form*.

## Biographical Information

*Ben Mills received his MSci from the School of Chemistry, University of Bristol, and continued with his PhD degree in the group of Professor Charl Faul working on the experimental and computational studies of aniline‐based materials. He completed his PhD in February 2017, and continued as PDRA in the Faul Research Group before finding employment in the public sector*.

## Biographical Information

*Kazuyoshi Watanabe received his B.Sc. and M.Sc. degrees from the Institute of Materials Science at University of Tsukuba in 2006 and 2008, and Ph.D. degree from the Department of Polymer Chemistry at Kyoto University under the supervision of Prof. Kazuo Akagi in 2015. His research interest is the self‐assembly of π‐conjugated oligomers and polymers, and their characteristic optoelectronic properties derived from their hierarchical ordered structures*.

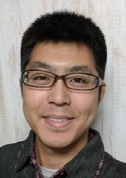

## Biographical Information

*Annela Seddon received her MChem from the University of Edinburgh in 1999, after which she undertook a PhD at the University of Bristol with Professor Stephen Mann FRS. From 2003–2006 she worked for Professor Paula Booth in the School of Biochemistry at the University of Bristol, before being awarded an EPSRC Life Sciences Interface Fellowship. From 2006–2009, she held this fellowship between Imperial College London and the University of Chicago. In 2009 she returned to the University of Bristol as the Graduate Teaching and Research Fellow within the Bristol Centre for Functional Nanomaterials (BCFN). In 2015, she was made Senior Lecturer in the School of Physics, and Director of the BCFN. Her research interests are in the study of self‐assembly of soft matter, and x‐ray scattering techniques that can be used to characterise such materials*.

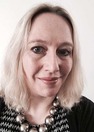

## Biographical Information

*Charl F. J. Faul is Professor of Materials Chemistry and Director of Graduate Recruitment for the School of Chemistry, University of Bristol, UK. He received his PhD from the University of Stellenbosch, South Africa, in 2000. After 4 years, first as post‐doctoral researcher, and then as senior scientist at the Max Planck Institute of Colloids and Interfaces (Potsdam, Germany), he moved to Bristol in 2005. He has held visiting professorships at the Helsinki University of Technology (2006–2010), the Chinese Academy of Sciences (National Centre for Nanoscience and Technology, Beijing, 2012) and is Adjunct Professor at the Department of Chemistry, Tsinghua University, Beijing, since November 2013. He is also co‐PI and on the management team of the BCFN. He leads a multi‐disciplinary materials chemistry research group, exploring the design principles and applications of novel redox‐active functional materials*.